# Biochemical characterization of the xylan hydrolysis profile of the extracellular endo-xylanase from *Geobacillus thermodenitrificans* T12

**DOI:** 10.1186/s12896-017-0357-2

**Published:** 2017-05-18

**Authors:** Martinus J.A. Daas, Patricia Murciano Martínez, Antonius H.P. van de Weijer, John van der Oost, Willem M. de Vos, Mirjam A. Kabel, Richard van Kranenburg

**Affiliations:** 1 Laboratory of Microbiology, Wageningen University, Stippeneng 4, Wageningen, 6708 WE The Netherlands; 2 Laboratory of Food Chemistry, Wageningen University, Bornse Weilanden 9, Wageningen, 6708 WG The Netherlands; 3 Corbion, Arkelsedijk 46, Gorinchem, 4206 AC The Netherlands

**Keywords:** *Geobacillus*, Thermophile, Endo-xylanase, Xylan, Biotechnology

## Abstract

**Background:**

Endo-xylanases are essential in degrading hemicellulose of various lignocellulosic substrates. Hemicellulose degradation by *Geobacillus* spp. is facilitated by the hemicellulose utilization (HUS) locus that is present in most strains belonging to this genus. As part of the HUS locus, the *xynA* gene encoding an extracellular endo-xylanase is one of the few secreted enzymes and considered to be the key enzyme to initiate hemicellulose degradation. Several *Geobacillus* endo-xylanases have been characterized for their optimum temperature, optimum pH and generation of degradation products. However, these analyses provide limited details on the mode of action of the enzymes towards various substrates resulting in a lack of understanding about their hydrolytic potential.

**Results:**

A HUS-locus associated gene (*GtxynA1*) from the thermophile *Geobacillus thermodenitrificans* T12 encodes an extracellular endo-xylanase that belongs to the family 10 glycoside hydrolases (GH10). The *GtxynA1* gene was cloned and expressed in *Escherichia coli*. The resulting endo-xylanase (termed *Gt*XynA1) was purified to homogeneity and showed activity between 40 °C and 80 °C, with an optimum activity at 60 °C, while being active between pH 3.0 to 9.0 with an optimum at pH 6.0. Its thermal stability was high and *Gt*XynA1 showed 85% residual activity after 1 h of incubation at 60 °C. Highest activity was towards wheat arabinoxylan (WAX), beechwood xylan (BeWX) and birchwood xylan (BiWX). *Gt*XynA1 is able to degrade WAX and BeWX producing mainly xylobiose and xylotriose. To determine its mode of action, we compared the hydrolysis products generated by *Gt*XynA1 with those from the well-characterized GH10 endo-xylanase produced from *Aspergillus awamori (Aa*XynA). The main difference in the mode of action between *Gt*XynA1 and *Aa*XynA on WAX is that *Gt*XynA1 is less hindered by arabinosyl substituents and can therefore release shorter oligosaccharides.

**Conclusions:**

The *G. thermodenitrificans* T12 endo-xylanase, *Gt*XynA1, shows temperature tolerance up to 80 °C and high activity to a variety of xylans. The mode of action of *Gt*XynA1 reveals that arabinose substituents do not hamper substrate degradation by *Gt*XynA1. The extensive hydrolysis of branched xylans makes this enzyme particularly suited for the conversion of a broad range of lignocellulosic substrates.

**Electronic supplementary material:**

The online version of this article (doi:10.1186/s12896-017-0357-2) contains supplementary material, which is available to authorized users.

## Background

Many *Geobacillus* species contain a hemicellulose utilization (HUS) locus in their genome that encodes for multiple hydrolytic enzymes and sugar transporters responsible for the degradation of hemicellulose [1, 2]. The most well characterized HUS locus belongs to *G. stearothermophilus* T-6 and its proposed degradation mechanism is based on a limited number of secreted hydrolytic enzymes and further processing of the generated degradation products intracellularly [3]. One of these secreted hydrolytic enzymes is an endo-xylanase that cleaves the xylan backbone of hemicellulose into shorter oligosaccharides [3, 4].

The xylan backbone is composed of xylosyl residues linked by ß-(1 → 4)-glycosidic bonds, and is typically decorated with a varying degree of substitutions containing (4-*O*-methyl)-α-d-glucuronic acids, α-l-arabinofuranosyl residues and acetyl residues [5]. The degree of substitution and polymerization is dependent on the origin of the substrate. Substrates from grasses and annual plants are rich in arabinoxylans, whereas hardwoods mostly consist of (4-*O*-methyl)-α-d-glucuronoxylan. This variety of substitutions requires multiple enzymes acting in synergy to fully degrade the xylans. Endo-(1 → 4)-ß-d-xylanases (E.C. 3.2.1.8) hydrolyse the ß-(1 → 4)-xylosidic bonds to release xylose and (substituted) xylo-oligosaccharides of various lengths that can be degraded further by other hemicellulases.

Endo-xylanases belonging to glycoside hydrolase family 10 and 11 are the best characterized and described [6]. Endo-xylanases belonging to family 10 (GH10) can tolerate the presence of substituents, such as arabinosyl residues, better than those belonging to glycoside hydrolase family 11 (GH11) [7]. An example of the mode of action of two endo-xylanases, belonging to families GH10 and GH11, from *Aspergillus awamori* (*Aa*) towards an arabinoxylan model substrate has been described [8]. The *Aa*GH10 was found to be able to cleave the xylan backbone next to a xylosyl residue substituted with one or two arabinosyl residues. In contrast, xylanases from family GH11 are hindered by arabinosyl substituents and cleave the xylan backbone only next to an unsubstituted xylosyl residue, producing longer oligosaccharides in comparison to those obtained from GH10 [8].

Endo-xylanases from *Geobacillus* species belong to the GH10 family and have been described in multiple studies [9–16]. These studies mainly focused on the characterization of activity ranges at higher temperatures and thermo-stability but lack detailed analyses of the mode of action of the enzyme on its substrate. Although some studies have described xylo-oligosaccharides formation upon hydrolysis of xylan by the endo-xylanase, the position of hydrolysis and the possible hindrance of substituents present on the xylan backbone have not been characterized [9, 12, 14, 16].

In this paper, we describe the cloning and overproduction in *E.coli* of *Gt*XynA1, a GH10 family endo-xylanase isolated from *Geobacillus thermodenitrificans*. We also determined the enzyme’s mode of action by identifying the generated hydrolysis products and propose a model for the degradation of substituted xylans.

## Methods

### Media, strains and cultivation methods

Wheat arabinoxylan (WAX) was of medium viscosity and supplied by Megazyme (Wicklow, Ireland). The chemical composition of this type of WAX has previously been described [17]. Beechwood xylan (BeWX) and all chemicals were purchased from Sigma-Aldrich (St Louis, MO, USA) unless otherwise specified. *Aa*XynA was kindly provided by the laboratory of Food Chemistry (Wageningen University) as described elsewhere [8].

Luria-Bertani (LB) medium contained per L: 10 g NaCl, 10 g tryptone (Oxoid), 5 g yeast extract (Roth). LB2 contains per liter: 10 g tryptone (Oxoid), 5 g yeast extract (Roth), 10 g sodium chloride and salts mix consisting of 1 g NH_4_Cl; 3 g NaCl; 1.50 g Na_2_SO_4_; 0.08 g NaHCO_3_; 1 g KCl; 1.8 g MgCl_2_ × 6H_2_O; 0.30 g CaCl_2_ × 2H_2_O. pH was set to 6.6 at room temperature and the medium was autoclaved for 20 min at 121 °C, after which 1 mL K_2_HPO_4_ (250 g/L) was added.

For plasmid propagation and protein expression *E. coli* strains DH5α and BL21(DE3) were used respectively. *E. coli* strains were grown in LB medium at 37 °C, pH 7.0 with an agitation speed of 150 RPM.

*Geobacillus thermodenitrificans* T12 was isolated from compost [18]. Strain T12 was grown in LB2 medium at 65 °C, 150 RPM and pH 7.0.

### Cloning of *Gt*XynA1

Genomic DNA of strain T12 was obtained using the MasterPure™ Gram Positive DNA Purification Kit (Epicentre) according to manufacturer’s protocol. The XynA coding sequence was cloned without the signal peptide, which was determined by SignalP4.1 to consist of the first 28 amino acids of the protein. Primers used for amplification of the *xynA* gene (GenBank accession number KX962565) were XynA-FW (5’-GCGCTCATGAAAACTGAACAATCATACGCTAAAAAG-3’) and XynA-RV (5’-GCGCCTCGAGCTTATGATCGATAATAGCCCAATACG-3’) that contain the *Bsp*HI and *Xho*I restriction sites, respectively. PCR amplification was performed on 50 ng of genomic DNA using PhusionHF DNA polymerase (Thermo Scientific) in the following conditions; 1 cycle 98 °C 5 min., 30 cycles of 98 °C 30 s, 50 °C 30 s and 72 °C 45 s followed by a final elongation step at 72 °C for 5 min. For enzyme characterization studies *Gt*XynA1 was cloned into pET24d(+) using the restriction sites of *Bsp*HI and *Xho*I. The reverse primer was designed in such a way that the stop codon was removed to include the His-tag from the pET24d vector. Plasmids were purified from *E. coli* DH5α using the GeneJET™ Plasmid Miniprep Kit (Thermo Scientific) and subsequently transformed into *E. coli* BL21(DE3).

### Overproduction and purification of *Gt*XynA1

Protein production was induced by adding 0.1 mM IPTG to a 250 mL culture at the start of inoculation. Culture was provided with 50 μg/mL kanamycin as antibiotics and incubated O/N at 37 °C at an agitation speed of 150 RPM. Cells were then centrifuged (4000×*g*, 15 min, 4 °C) and cell pellet was resuspended in 25 mL phosphate buffer at a pH of 7.4. Cell suspension was disrupted using French Press and cell free extract (CFE) was obtained by centrifugation (30,000×*g*, 20 min, 4 °C). Filtered CFE was applied to a HisPrep FF 16/10 Ni-NTA Sepharose column (GE Healthcare) equilibrated with 50 mM sodium phosphate buffer (pH 7.4) containing 300 mM NaCl. Bound *Gt*XynA1 was eluted using a linear gradient of 0–500 mM imidazole in the same buffer. Purified *Gt*XynA1 was then desalted by applying the protein solution to a HiPrep 26/10 desalting column (GE Healthcare) equilibrated with 50 mM sodium phosphate buffer (pH 7.4). Recombinant xylanase was eluted at a rate of 5 mL/min and stored at−20 °C for further research. Protein purity was assayed by SDS-PAGE analysis using a 10% (*w/v*) polyacrylamide gel according to the method of [19]. A PageRuler™ protein ladder was used and bands were visualized using PageBlue™ Protein Staining Solution (Thermo Scientific).

### Enzyme activity assays

Wheat arabinoxylan (WAX), Beechwood xylan (BeWX) and Birchwood xylan (BiWX) were incubated with the purified *Gt*XynA1 in 10 mM NaOAc buffer, pH 6.0 (1 mL, 10 mg substrate dry matter) at 60 °C for 24 h. The enzymes were dosed at 0.1% (w/w) protein based on substrate dry matter. Next, 2 μl of 4 M HCl was added to stop the enzyme activity and the sample was centrifuged (10,000×*g*, 10 min, 10 °C) prior to analysis.

The pH and temperature optima were tested by incubating WAX with *Gt*XynA1 in a range of pH 3 to 9. For the pH range of 3 to 8 we used 50 mM sodium citrate buffer and for the pH range of 8 to 10 we used 200 mM Tris–HCl buffer. Buffers were adjusted for correct pH at the temperature of incubation. Optimum temperature was determined in a range from 30 to 100 °C. Incubation time, enzyme dosage and stopping the reaction were as described above.

All samples collected were submitted to the PAHBAH-assay, HPSEC, HPAEC and RP-UHPLC-UV-MS.

### Analysis of hydrolysis products

The supernatants obtained were analysed for the amount of reducing ends present by the PAHBAH reducing assay in duplicate [20].

### HPSEC

High performance size exclusion chromatography (HPSEC) of WAX and BiWX before and after incubation with *Gt*XynA1 was performed on an Ultimate 3000 HPLC system (Thermo Scientific, Sunnyvale, CA, USA) equipped with a set of three TSK-gel columns (6.0 mm × 15.0 cm per column) in series (SuperAW4000, SuperAW3000, SuperAW25000, Tosoh Bioscience, Stuttgart, Germany) in combination with a PWX-guard column (Tosoh Bioscience). HPSEC was controlled by the Chromeleon software (Thermo Scientific). Elution took place at 40 °C with 0.2 M sodium nitrate at a flow rate of 0.6 mL/min. The eluate was monitored using a refractive index (RI) detector (Shoko Scientific Co., Yokohama, Japan). Calibration was made by using pullulan series (Polymer Laboratories, Union, NY, USA) with a molecular weight in the range of 0.18–788 kDa.

### HPAEC

Oligosaccharides and monosaccharides released after enzymatic incubation of WAX and BeWX with *Gt*XynA1 were analysed by HPAEC as described elsewhere [21].

### RP-UHPLC-UV-MS

Xylo-oligosaccharides produced by the enzymes were 2-AA labelled and analysed by RP-UHPLC-UV-MS as previously described by Murciano Martinez et al. [21].

## Results

In this study, we cloned and overexpressed the *Geobacillus thermodenitrificans* T12 *xynA1* gene in *E.coli* and determined the mode of action of the *Gt*XynA1 xylanase enzyme on wheat arabinoxylan (WAX), beechwood xylan (BeWX) and birchwood xylan (BiWX). The *xynA* gene is 1224 bp long and encodes a protein of 407 amino acid residues, which belongs to GH family 10. The amino acid sequence contains a signal peptide of 28 amino acids and two catalytic residues were predicted at positions H264 and D295. Phylogenetic analysis revealed high amino acid identity to several *Geobacillus* endo-xylanases (Table 1).

**Table 1:** Sequence identity of several characterized endo-xylanases to the *G. thermodenitrificans* T12 endo-xylanase

Origin	DNA identity (%)	AA identity (%)	Reference
*G. thermodenitrificans* T12	-	-	This study
*G. thermodenitrificans* JK1	99	100	[11]
*G. thermodenitrificans* TSAA1	99	100	[16]
*G.* sp. TC-W7	99	99	[13]
*G.* sp. 71	91	91	[10]
*G. stearothermophilus* T-6	84	84	[4]

### Cloning, expression and characterization of *Gt*XynA1

**Fig. 1:**
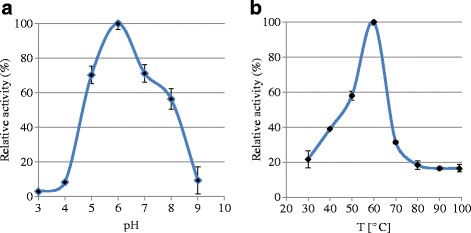
pH (**a**) and temperature (**b**) profiles of the incubated *Gt*XynA1 with wheat arabinoxylan (WAX)

**Fig. 2:**
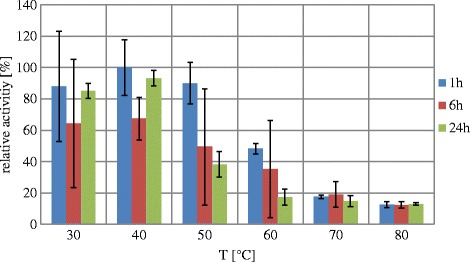
Temperature stability of *Gt*XynA1. The temperature stability was determined by incubating *Gt*XynA1 at temperatures between 30 and 80 °C for 1 h, 6 h, and 24 h after which the residual activity was measured against wheat arabinoxylan at 60 °C and a pH of 6.0

The gene coding for *Gt*XynA1 (1224 bp) was PCR-amplified, cloned into the pET24d vector and successfully expressed in *E.coli* BL21(DE3). The endo-xylanase was purified to apparent homogeneity, as it appeared as a 50 kDa single band on SDS-PAGE after visualization with PageBlue stain (Additional file 1: Figure S1; Additional file 2: Figure S2, lane 6). The purified endoxylanse *Gt*XynA1 showed highest activity at pH 6.0 in a broad activity range from pH 4.0 to 9.0 (Fig. 1a). *Gt*XynA1 showed activity between 40 °C to 80 °C with an optimum at 60 °C (Fig. 1b). The thermostability of *Gt*XynA1 was tested in the range of 30 °C to 80 °C. After 1 h at 50 and 60 °C the enzyme retained 90 and 45% residual activity, respectively, whereas the residual activity after 1 h at 70 °C was only 18% (Fig. 2).

### Mode of action

**Fig. 3:**
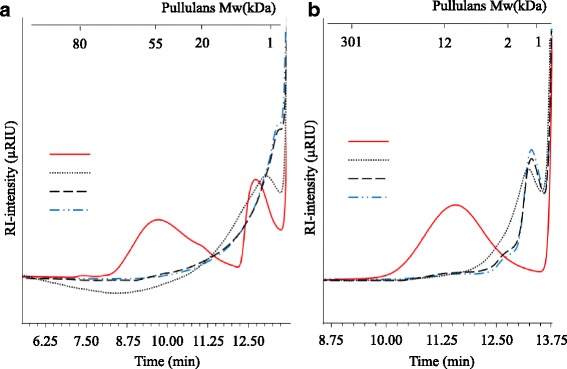
Molecular weight distributions of wheat arabinoxylan (WAX) (**a**) and beechwood xylan (BeWX) (**b**) incubated with *Gt*XynA1 for 0, 1, 6 and 24 h

**Fig. 4:**
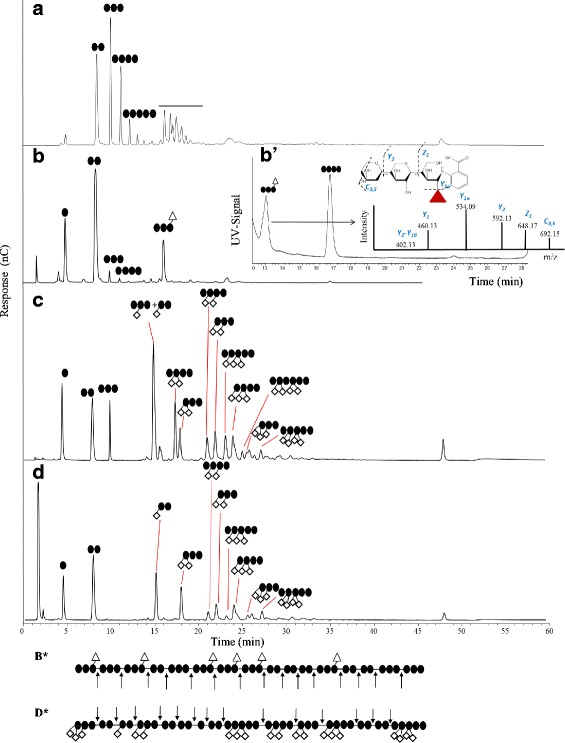
HPAEC elution pattern of with *Aa*XynA and *Gt*XynA1 incubated with beech wood xylan (BeWX) (**a**, **b** respectively) and wheat arabinoxylan (WAX) (c, d respectively) for 24 h, and respective cleavage pattern of GtXynA1 on BeWX (b*) and WAX (d*). • = xylosyl residue, ◊ = arabinosyl residue, ▲ = 4-*O*-methylglucuronic acid. (**b**) shows the identification of the main substituted xylo-oligosaccharide from B. The structure is identify based on its UV signal and the MS^2^ fragmentation pattern

Activity of *Gt*XynA1 towards wheat arabinoxylan (WAX), birchwood xylan (BiWX), and beechwood xylan (BeWX) was determined as outlined in materials and methods. Degradation profiles of BiWX and BeWX were similar (data not shown), therefore only the BeWX degradation patterns will be further explained. WAX contains β-(1 → 4) linked xylosyl residues substituted with α-(1 → 2) linked arabinosyl residues or with both α-(1 → 2) and α-(1 → 3) linked arabinosyl residues. BiWX and BeWX contain β-(1 → 4) linked xylosyl residues substituted with α-(1 → 2) linked 4-*O*-methylglucuronic acid (UA_me_). Both WAX and BeWX were incubated with *Gt*XynA1 at its optimum pH and temperature for 24 h (Fig. 3). Both WAX and BeWX were degraded to oligomers and monomers, as deduced from the disappearance of soluble polymers (WAX around 55 kDa, BeWX around 12 kDa; Fig. 3) and the increase of lower molecular weight products of around 1 kDa after 24 h incubation. The difference in molecular weight of the polymeric structures analysed at 0 h incubation is because WAX is completely soluble and BeWX is only partly soluble [22]. *Gt*XynA1 incubated with WAX and BeWX released a series of linear and substituted xylo-oligosaccharides that were identified based on the known HPAEC elution of WAX digested with the well-characterized GH10 endo-xylanase from *Aspergillus awamori* (*Aa*XynA) [8]. The oligomeric profile released by *Gt*XynA1 is comparable to the profile produced by *Aa*XynA, supporting that *Gt*XynA1 is a GH10 endo-xylanase (Fig. 4). Only one substituted oligosaccharide was produced from BeWX, eluting at 16 min (Fig. 4), which was identified as a xylotriose substituted by 4-*O*-methylglucuronic acid located at the non-reducing end (Fig. 4b’) after 2-AA labelling and analysis by UPLC-UV-MS. The UV chromatogram at 340 nm of 2-AA labelled degradation products from BeWX and the full MS spectra overlapped. The two predominant masses seen in the full MS spectra were 724 (m/z) and 666 (m/z) (data not shown), and they overlapped with UV chromatogram peaks eluting at 13 and 16.8 min, respectively. Fig. 4 also shows that *Gt*XynA1 is able to degrade WAX and BeWX to smaller-sized linear xylo-oligosaccharides, mainly xylose and xylobiose. This observation is substantiated on the integrated peak area of linear xylo-oligosaccharides (Table 2). On the contrary, *Aa*XynA is able to degrade WAX and BeWX producing mainly xylobiose and xylotriose, but also xylotetraose, xylopentaose and xyloheaxaose (Table 2).

**Table 2:** HPAEC integrated peak area of linear xylo-oligosaccharides produced from WAX and BeWX after 24 h incubation with *Gt*XynA1 and *Aa*XynA

Enzyme tested	*Gt*XynA1		*Aa*XynA	
Substrate	WAX	BeWX	WAX	BeWX
Quantification	peak area			
Xylose (●)	87.8	165.6	4.4	17
Xylobiose (●●)	192.7	457.9	17.5	157.1
Xylotriose (●●●)	*	15.5	10.6	131.7
Xylotetraose (●●●●)	*	6.9	4.3	81.1
Xylopentaose (●●●●●)	*	*	1.2	25.3
Xylohexaose (●●●●●●)	*	*	*	10.2

• = xylosyl residue; * = not detected

When comparing the diversity of substituted xylo-oligosaccharides produced (Fig. 4), it is clear that *Gt*XynA1 produces shorter oligomers from both WAX and BeWX compared to *Aa*XynA. Substituted xylo-oligosaccharides are identified in a range of DP from 2 to 6 by both HPAEC (Fig. 4a) and MS (not shown), suggesting that *Aa*XynA is less tolerant towards 4-*O*-methylglucuronic acid substituted xylan.

Degradation products formed during the degradation of WAX by *Aa*XynA that where eluting at 15, 17 and 25 min are absent in the profile of *Gt*XynA1 incubated on WAX (Fig. 4c, d). These xylo-oligosaccharides are characteristic because of the presence of two arabinosyl residues located at the *O*-2 and *O*-3 of the same xylosyl residue. Hence, *Gt*XynA1 cleaves next to an adjacent non-substituted residue, from the non-reducing end when a single substituted residue is present. In case the xylosyl residue is doubly substituted by arabinosyl residues, *Gt*XynA1 needs two adjacent unsubstituted residues at the non-reducing end. In case the cleavage takes place from the reducing end, the enzyme can cleave directly next to the substituted (single or doubly substituted) residue in most cases (Fig. 4d, minutes 18, 22, 25.5). However, when the substituted residue (single or doubly substituted), is followed by a doubly substituted xylosyl residue, the enzyme cleaves next to an unsubstituted residue (Fig. 4d, minutes 23, 27). On the contrary, the presence of more than one doubly or single substituted xylosyl residues obstructs the action of *Aa*XynA. Based on the information provided by the HPAEC chromatograms and RP-UV-UHPLC-MS profile on the linear and substituted xylo-oligosaccharides produced by the enzymes, an schematic mode of action of *Gt*XynA1 is proposed in Fig. 4b* and d*.

## Discussion

The ability of *Geobacillus* species to degrade xylan relies on the activity of the hemicellulose utilization (HUS) locus. The HUS-locus contains a variety of xylan degrading enzymes, sugar transporters and metabolic pathways for the complete utilization of (substituted) xylans. Xylan degradation initiates by the action of an extracellular endo-xylanase (XynA1) that cleaves the xylan backbone, thereby producing (substituted) xylo-oligosaccharides. The linear oligosaccharides generated by the action of XynA1 can then enter the cell through an ABC-transporter that is encoded by the *xynEFG* operon whereas the substituted oligosaccharides enter the cell *via* other substrate specific ABC transporters [1, 3, 23]. In this research, we cloned and expressed *Gt*XynA1, the endo-xylanase of *G.* *thermodenitrificans* T12 and analysed its mode of action.

The purified endo-xylanase *Gt*XynA1 showed highest activity at pH 6.0 in a broad activity range from pH 4.0 to 9.0 (Fig. 1a), which is in line with previous reports on endo-xylanases from geobacilli [11, 13, 16]. *Gt*XynA1 showed activity between 40 °C to 80 °C with an optimum at 60 °C (Fig. 1b) where previous reports on *Geobacillus* endo-xylanases demonstrated optimum temperatures from 60 °C up to 75 °C [11, 13, 14, 16]. The thermostability of *Gt*XynA1 was tested in the range of 30 °C to 80 °C. After 1 h at 50 and 60 °C the enzyme retained 90 and 45% residual activity, respectively, whereas the residual activity after 1 h at 70 °C was only 18% (Fig. 2). The endo-xylanase characterized from *Geobacillus stearothermophilus* T-6 remains 90% active (1 h time point) after incubation at 70 °C [4]. This higher temperature stability of the T-6 endo-xylanase may be caused by its production from the native strain instead of a recombinant expression system in *E.coli*. A lower thermostability of an endo-xylanase produced in *E. coli* compared to the same protein produced in *Geobacillus* has been reported for *Geobacillus* sp. TC-W7 [13], which produces an endo-xylanase highly similar to *Gt*XynA1 (Table 1). Alternatively, the difference in thermostability between *Gt*XynA1 and the T-6 endo-xylanase may be (partly) caused by differences in their amino acid composition. The *Gt*XynA1 enzyme was more thermostable in comparison with other bacterial endo-xylanases such as XynC from *Bacillus subtilis* 168, that shows around 20% residual activity at 50 °C and no residual activity at 60 °C [24]. The thermostability of *Gt*XynA1 has great advantage for the degradation of xylan at higher temperatures (60 °C) over enzymes produced by most mesophilic organisms.

In previous studies, *G. stearothermophilus* T-6 was grown on 4-*O*-methyl –d-glucurono-d-xylan and the main end products found were xylotriose substituted with 4-O-methyl–d-glucuronic acid together with xylobiose and xylose [25]. Although we found the same end products in the present study, we also detected xylotriose and xylotetraose as minor end products (Fig. 4). Only one substituted oligosaccharide was produced, identified as a xylotriose substituted by 4-*O*-methylglucuronic acid located at the non-reducing end (Fig. 4b’).

**Fig. 5:**
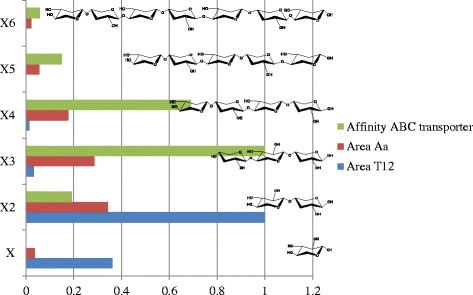
Peak area ratio of linear end products after 24 h incubation on beech wood xylan in comparison to oligosaccharide transporter (*xynEFG*) affinity. Green: ratio of affinity of the XynEFG oligosaccharide transporter of *G. stearothermophilus* towards different oligosaccharides. Red: ratio of the end product area after a 24 h incubation of *Aa*XynA on beechwood xylan. Blue: ratio of the end product area after a 24 h incubation of *Gt*XynA1 on beechwood xylan

When comparing the mode of action on WAX, the main difference between *Gt*XynA1 and *Aa*XynA is that the former enzyme is less hindered by arabinosyl substituents. This is deduced from the difference in degradation products formed between the incubations of *Gt*XynA1 and *Aa*XynA on WAX. Similar end products were found in previous studies using TLC analysis but, no quantification of the linear end products and no identification of substituted end products was performed [14, 26]. Formed xylo-oligosaccharides are taken up by the cell *via* specific sugar transporters [3]. These transporters are encoded by the operon *xynEFG*, located in the HUS locus, and have a preference for short xylo-oligosaccharides within a DP of 2 to 6. More specifically, highest affinity is towards trisaccharides and for other xylo-oligosaccharides the affinity is as follows: *X*2 < X3 > X4 > X5 > X6 [1]. The relative quantity of different xylo-oligosaccharides formed by the action of *Gt*XynA1 on BeWX shows that mostly xylobiose and xylose is formed (Table 2, Fig. 5). The discrepancy between end products formed by *Gt*XynA1 and the affinity of the sugar transporter XynEFG is most likely caused by the 24 h incubation as *Gt*XynA1 is also active towards xylotriose. Previous studies demonstrated the formation of xylotriose within 30 min of incubation and prolonged incubation resulted in a decrease of xylotriose and an increase in xylobiose and xylose [14, 26].

The mode of action of *Gt*XynA1 creates linear and branched xylo-oligosaccharides of which most are in the DP range of 2 to 6. These small branched oligosaccharides are believed to enter the cell *via* specific ABC-transporters [3]. For most of these transporters their affinity towards linear oligosaccharides was determined [1, 23, 25]. However, for the arabino-oligosaccharide transporter AbnEFJ it was shown that the binding constants of branched oligosaccharides where 2 orders of magnitude higher than those obtained for linear oligosaccharides [23]. It is likely that also other ABC transporters that are involved in the uptake of oligosaccharides have higher affinity for branched oligosaccharides in comparison to linear oligosaccharides. The possibility of transporting branched oligosaccharides enables the *Geobacillus* species to use natural substrates in a rapid and efficient way.

## Conclusions

*Gt*XynA1 is a GH10 endo-xylanase isolated from *G. thermodenitrificans* T12 and is active towards WAX and BeWX, showing extensive degradation of the soluble polymeric structures to linear xylo-oligosaccharides in a DP range from 1 to 4 and to relatively short substituted xylo-oligosaccharides. These degradation products are taken up by the cells *via* specific ABC transporters. We have demonstrated that *Gt*XynA1 is able to cleave the xylan backbone of WAX next to an arabinose substituent on the reducing end and the need for one to two free xylose units on the non-reducing end. In contrast, the hydrolysis of BeWX by *Gt*XynA1 occurs next to a 4-*O*-methylglucuronic acid located at the non-reducing end. The thermophilic origin and its extensive degradation of xylans from different origins demonstrates the potential of *Gt*XynA1 for biomass conversion processes at elevated temperatures.

## Additional files

Additional file 1: Figure S1. FPLC purification of *Gt*XynA1. Protein fraction not bound to the nickel column eluted with the first 65 mL eluent. Bound *Gt*XynA1 protein was removed from the nickel column using a imidazole gradient (yellow line) which increased from 0 mM to 500 mM over a time span of 20 min (A). Fractions 22–25 were pooled and used for desalting the purified *Gt*XynA1 (B). Fractions 8–10 of the desalting column were pooled and then used for further experiments. (DOCX 311 kb)

Additional file 2: Figure S2. 10% SDS-PAGE of purified endo-xylanase from *G. thermodenitrificans* T12 followed by PageBlue staining. Lane 1: Protein marker; Lane 2: Pellet fraction; Lane 3: cell-free extract; Lane 4: non-binding protein fraction from FPLC; Lane 5: Purified recombinant *Gt*XynA1; Lane 6: Purified and desalted recombinant *Gt*XynA1; Lane 7: Protein marker. (DOCX 614 kb)

## Abbreviations

GH: Glycosyl hydrolase
SP: Signal peptide
HPSEC: High Pressure Size Exclusion Chromatography
HPAEC: High-Performance Anion-Exchange Chromatography
MALDI-TOF MS: Matrix-Assisted Laser Desorption Ionization – Time Of Flight Mass Spectrometry
BeWX: BeechWood glucuronoXylan
BiWX: BirchWood glucuronoXylan
CFE: Cell free extract
DP: Degree of Polymerization
UHPLC-UV-MS: Ultra High Performance Liquid Chromatography Ultra Violet Mass Spectrometry
2-AA: Anthranilic Acid.
