# Supplementary material for: Biochemical characterization of the xylan hydrolysis profile of the extracellular endo-xylanase from Geobacillus thermodenitrificans T12
Source: BMC Biotechnol. 2017 May 18;17:44. doi: 10.1186/s12896-017-0357-2 (PMC5437666; doi:10.1186/s12896-017-0357-2)
Supplement: Supplementary file 1 — FPLC purification of GtXynA1. Protein fraction not bound to the nickel column eluted with the first 65 mL eluent. Bound GtXynA1 protein was removed from the nickel column using a imidazole gradient (yellow line) which increased from 0 mM to 500 mM over a time span of 20 min (A). Fractions 22–25 were pooled and used for desalting the purified GtXynA1 (B). Fractions 8–10 of the desalting column were pooled and then used for further experiments. (DOCX 311 kb) [file 12896_2017_357_MOESM1_ESM.docx]

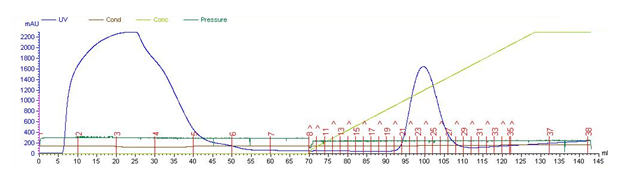

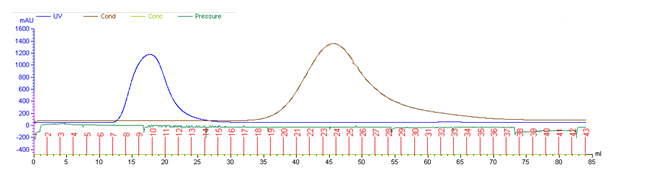


A

B

Fig. S1 FPLC purification of *Gt*XynA1. Protein fraction not bound to the nickel column eluted with the first 65 mL eluent. Bound *Gt*XynA1 protein was removed from the nickel column using a imidazole gradient (yellow line) which increased from 0 mM to 500 mM over a time span of 20 minutes (A). Fractions 22-25 were pooled and used for desalting the purified *Gt*XynA1 (B). Fractions 8-10 of the desalting column were pooled and then used for further experiments.
