# Supplementary material for: Biochemical characterization of the xylan hydrolysis profile of the extracellular endo-xylanase from Geobacillus thermodenitrificans T12
Source: BMC Biotechnol. 2017 May 18;17:44. doi: 10.1186/s12896-017-0357-2 (PMC5437666; doi:10.1186/s12896-017-0357-2)
Supplement: Supplementary file 2 — 10% SDS-PAGE of purified endo-xylanase from G. thermodenitrificans T12 followed by PageBlue staining. Lane 1: Protein marker; Lane 2: Pellet fraction; Lane 3: cell-free extract; Lane 4: non-binding protein fraction from FPLC; Lane 5: Purified recombinant GtXynA1; Lane 6: Purified and desalted recombinant GtXynA1; Lane 7: Protein marker. (DOCX 614 kb) [file 12896_2017_357_MOESM2_ESM.docx]

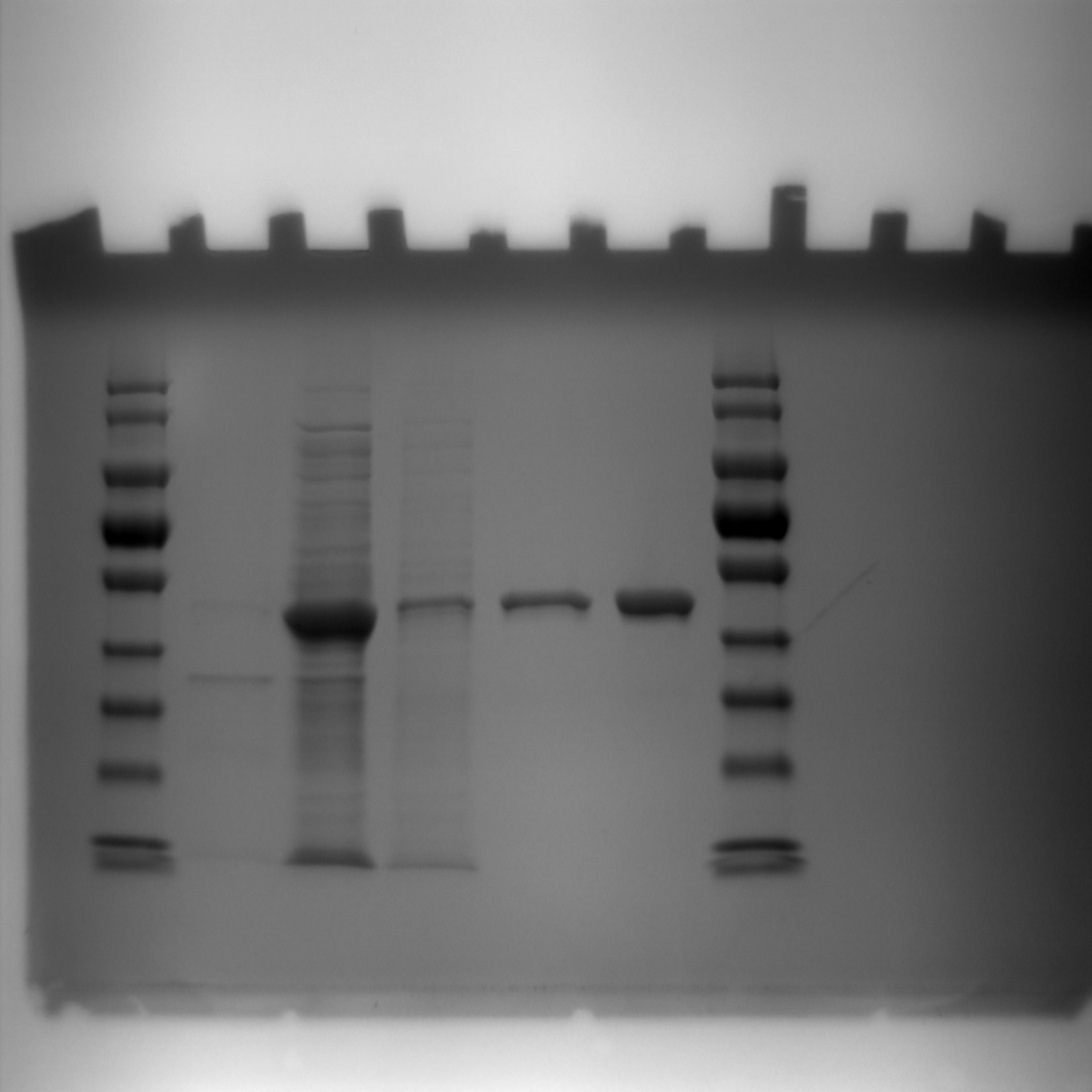


**40 -**

**180 -**

**kDa**

**55 -**

**70 -**

**130 -**

**100 -**

**35 -**

**25 -**

**10 -**

**4**

**5**

**6**

**3**

**2**

**1**

**7**

Fig. S2 10% SDS-PAGE of purified endo-xylanase from *G. thermodenitrificans* T12 followed by PageBlue staining. Lane 1: Protein marker; Lane 2: Pellet fraction; Lane 3: cell-free extract; Lane 4: non-binding protein fraction from FPLC; Lane 5: Purified recombinant *Gt*XynA1; Lane 6: Purified and desalted recombinant *Gt*XynA1; Lane 7: Protein marker
